# Virtual Screening-Based Drug Development for the Treatment of Nervous System Diseases

**DOI:** 10.2174/1570159X20666220830105350

**Published:** 2023-09-25

**Authors:** Qian Li, Zhaobin Ma, Shuhua Qin, Wei-Jiang Zhao

**Affiliations:** 1 Wuxi School of Medicine, Jiangnan University, Wuxi 214122, Jiangsu, P.R. China;; 2 College of Life Science and Technology, Kunming University of Science and Technology, Kunming 650504, Yunnan, P.R. China;; 3 Department of Cell Biology, Wuxi School of Medicine, Jiangnan University, Wuxi 214122, Jiangsu, P.R. China

**Keywords:** Virtual screening, molecular docking, nervous system diseases, neuronal reprogramming, small molecular compound, drug design

## Abstract

The incidence rate of nervous system diseases has increased in recent years. Nerve injury or neurodegenerative diseases usually cause neuronal loss and neuronal circuit damage, which seriously affect motor nerve and autonomic nervous function. Therefore, safe and effective treatment is needed. As traditional drug research becomes slower and more expensive, it is vital to enlist the help of cutting-edge technology. Virtual screening (VS) is an attractive option for the identification and development of promising new compounds with high efficiency and low cost. With the assistance of computer-aided drug design (CADD), VS is becoming more and more popular in new drug development and research. In recent years, it has become a reality to transform non-neuronal cells into functional neurons through small molecular compounds, which provides a broader application prospect than transcription factor-mediated neuronal reprogramming. This review mainly summarizes related theory and technology of VS and the drug research and development using VS technology in nervous system diseases in recent years, and focuses more on the potential application of VS technology in neuronal reprogramming, thus facilitating new drug design for both prevention and treatment of nervous system diseases.

## 1 INTRODUCTION

The development of a new drug is a long and costly process, usually lasting about ten years of time at the cost of billions of dollars [1]. The key to a successful new drug research and development project is often the determination of selected compounds in the initial stage of research. The rapid development of genomics and proteomics has improved the understanding of diseases, greatly changed the thinking of new drug research and development, and formed the research strategy of “from gene to the drug” [2]. This requires us to study the role of drugs at gene, transcription and protein levels. However, quickly finding the entry point of drug design research, based on which drug activity and optimization, and drug action targets and mechanisms can be explored, is a key step for medical researchers.

The discovery of drugs has experienced a transformation from empirical experiments to scientific research. In the process of drug development, it is not ethically and practically feasible to test the activity of all candidate compounds in human or animal models of human disease [3]. Recently, with the integration of computer science and medical research, the drug development technology represented by high-throughput screening (HTS) has opened a new chapter of modern drug discovery [4]. The combination of microscreening systems and highly automated operations has expanded the scale of drug screening and greatly promoted the process of drug research and development. However, the hit rate of HTS is relatively low. This is mainly due to the high proportion of false positivity in the screening results, the non-specific aggregation of small molecules and the solubility of compounds. This low hit rate limits the use of HTS for research projects to screen large compound libraries. The development of synthetic chemistry also provides a wealth of compounds to be tested for HTS. However, completely relying on HTS at the initial stage of drug screening is still a very huge and expensive project, which requires exploration of a new method to optimize this process. Recently, computerized automatic filtering of huge compound libraries has emerged as a promising project. Virtual screening (VS) is a natural extension of this work, which can reduce the initial cost of identifying drug targets and improve the opportunity to find ideal drug candidates [5].

## 2 VIRTUAL SCREENING

**Fig. (1):**
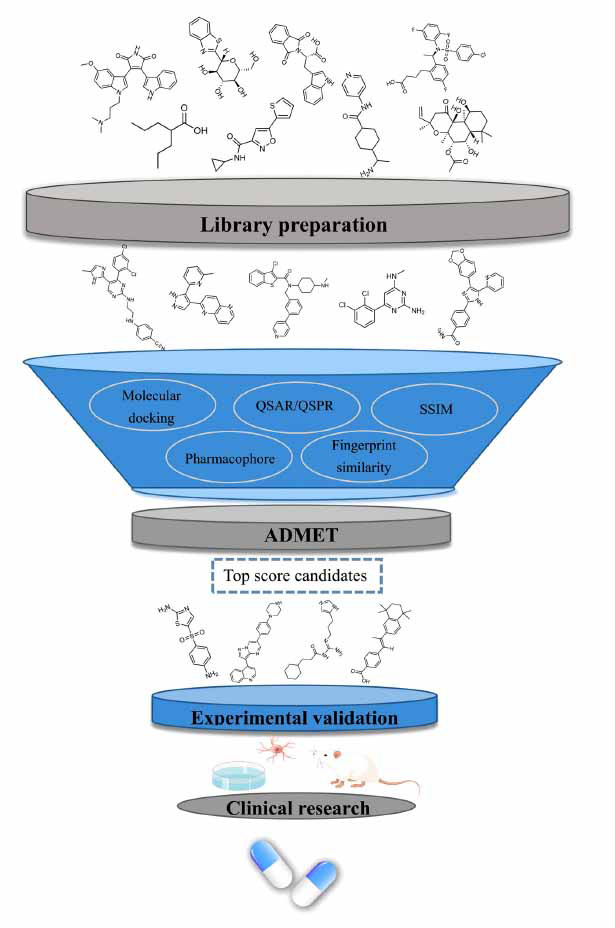
The workflow demonstrating the process of virtual screening. Some materials in this Figure are drawn by the FigDraw platform.

Virtual screening, also known as computer screening, was established in the late 1990s. By using the high-speed computing performance of the computer, compounds that are more likely to combine with specific biological targets are screened from a large number of candidate compounds, so as to effectively improve the success rate of discovering potential active compounds [6]. In most cases, VS is stacked up in the form of a workflow, with the sequential combination of different methods. These methods help filter unwanted compounds, narrow the scope and determine a group of compounds with potential biological activities. The compounds studied do not necessarily exist during the process of screening, and the “test” does not consume valuable substances. Compounds that survive in all VS filters are called hit compounds, which must be subsequently evaluated in the laboratory to confirm their biological activity [7]. The basic process of VS mainly includes [8]: 1) preparing appropriate compounds database; 2) selecting the appropriate screening method in regard to the availability of the receptor structure; 3) analyzing the pharmacokinetic properties of candidate compounds, and 4) performing biological experimental tests on the screening results (Fig. **1**). Molecular docking is the major structure-based virtual screening (SBVS) process, which mainly depends on the structural content of a target protein to calculate its interaction energy with small molecular compounds. If the high-resolution structural data of a protein is available, SBVS is the first choice. Ligand-based virtual screening (LBVS) is relatively cheap in calculation, because macromolecules are not involved in the calculation. For this reason, it is usually used in the early stage of VS workflow process. The success of existing LBVS methods depends on a large number of data samples. When the information of known active ligand samples is insufficient, it is difficult to obtain good prediction performance with LBVS.

### 2.1 Virtual Library Preparation

The selection of a virtual library (VL) that fits the screening requirements is the first and most important stage in performing VS. VS research is heavily reliant on the quality of the VL to ensure the identification of the most promising or bioactive compounds [9]. The quality of VL is determined by two primary criteria: diversity and representativeness, in addition to the quantity of chemicals covered. Diversity can ensure that all selected molecules are as different from each other as possible, so as to cover as much chemical property space as possible. Representativeness means that the distribution of compounds in chemical space is similar to that in the VL during sampling [10]. A good VL has well-planned structural data and good data set preparation, which can be directly used in VS research. There are a variety of compound libraries that are commonly employed in VS research (Table **1**).

### 2.2 Ligand-Based Virtual Screening (LBVS)

The criteria used to define similarity in the literature include [11]: 1) compounds that share at least one functional group or have a similar chemical skeleton; 2) compounds that have similar three-dimensional shapes or pharmacophore models; and 3) similar descriptors or properties of the compounds. The research idea of LBVS is that compounds with comparable structure and chemical characteristics may have similar biological effects. LBVS can predict the activity of compounds by establishing a common pharmacophore, QSAR model or structural similarity method after analyzing a series of known ligand structures, physical and chemical properties and activity relationships. Fig. (**2**) shows a method for identifying a similar 2D skeleton.

#### 2.2.1 Pharmacophore Model

Lemon Kier first proposed the modern definition of “pharmacophore” in 1967, and it first appeared in publications in 1971 [12]. As stated in a more specific official IUPAC definition, “a pharmacophore is the ensemble of steric and electronic features that is necessary to ensure the optimal supra-molecular interactions with a specific biological target structure and to trigger (or to block) its biological response” [13]. The VS based on the pharmacophore model makes maximum use of the existing structural information of small-molecule ligands. It can be assumed that ligand molecules with different structures with similar pharmacodynamic patterns can be recognized by the same binding site of a biological target, thus showing similar biological profiles [14].

The pivotal procedure of the pharmacophore model is to identify their key common features and relative directions (also known as pharmacophore localization) from a group of active molecules. An important condition for its success is that the active ligand binds to the same receptor in the same direction at the same binding site. Otherwise, the generated pharmacophore model cannot accurately describe the mode of action [15]. A well-constructed pharmacophore model should cover as many active chemicals as possible while excluding inactive ones. It is also required to assess the pharmacophore model ability with a test set and bait set [16]. Usually, multiple pharmacophore model ability can be extracted and sorted according to the appropriate fitness function. There are some programs on the market that can automatically generate pharmacophore models, including Discovery Studio (DS), LigandScout, Sybyl, MOE and so on. Fig. (**3**) shows a ligand-based pharmacophore model that may promote the transdifferentiation of astrocytes.

#### 2.2.2 Quantitative Structure-Activity/Property Relationship (QSAR/QSPR)

Quantitative structure-activity relationship (QSAR) is a chemical data analysis tool created by Corwin Hansch [17]. With the in-depth study of QSAR, its modeling was used to evaluate the pharmacokinetic properties of compounds. It has now been developed into quantitative structure-property relationship (QSPR) models [18]. QSAR/QSPR models are developed by establishing empirical, linear or nonlinear relationships between molecular descriptors and experimentally measured properties or biological activities of these molecules, and then by applying these models to predict or design new compounds with required properties [19]. In recent years, scientists have used different methods to establish a reliable QSAR/QSPR model, including molecular modeling, pattern recognition, machine learning or artificial intelligence [20], which extends the traditional boundaries of the model. The accumulated experience and best practices in data management, model development and verification of QSAR/QSPR models provide valuable guidance for many fields [21].

The process of creating QSAR/QSPR models can be divided into four parts. The initial step is to collect structural and biological activity or pharmacokinetic data. Although it is advised that the number of compounds in the training set be as high as possible, the compound number in the training set can be changed depending on the system's complexity [22]. The second step is to calculate chemical descriptors at various representation levels. The information contained by the descriptor is usually determined by the type of molecular representation used and the computation algorithm used [23]. The third stage is to link the descriptors gained with biological activity or pharmacokinetic data so as to create a mathematical model for screening [24]. The fourth phase is verification, evaluation, and application of QSAR/QSPR models.

#### 2.2.3 Structural Similarity Method (SSIM)

The structural similarity method is a method of similarity matching through various molecular descriptors or molecular fingerprints, so as to identify whether the compounds have a similar activity or disease treatment mechanism [25]. With the development of drug analysis technology, more and more molecular fingerprinting methods have emerged. The molecular descriptor system can be classified into either two-dimensional descriptors or three-dimensional descriptors. Due to the strong characteristics and fast calculation speed, 2D molecular descriptors have become a common method in LBVS [26]. The 2D molecular descriptors consist of many binary bits, their structural fragments are usually generated by the fragment method. Fig. (**4**) shows the 2D-substructure fingerprints of levodopa and carbidopa. The commonly used fragment methods include MDL search keys [27], Daylight fingerprints [28] and Atom center fragments (ACFs) [29]. Because the binding force between molecules and target proteins depends on atomic interaction in 3D space, 3D descriptors are a superior option. However, a single molecule usually has many 3D representations, making the 3D model more demanding in storage space and computing time. Although 2D descriptors function well, they disregard properties linked to molecular spatial configuration. In contrast, 3D descriptors correctly consider these characteristics when evaluating similarity [30]. The best method is to use both techniques and verify the results through subsequent higher precision algorithms.

### 2.3 Structure-Based Virtual Screening (SBVS)

SBVS plays a crucial role in finding novel compounds and exploring new action modes. It usually uses molecular docking technology to predict the main binding modes between ligand molecules and known 3D proteins according to the principles of spatial matching and energy matching [31]. After clarification of disease-related drug target information and crystal structure, SBVS strategy can become the preferred technology for drug research and development without exception.

#### 2.3.1 Molecular Docking

**Fig. (5):**
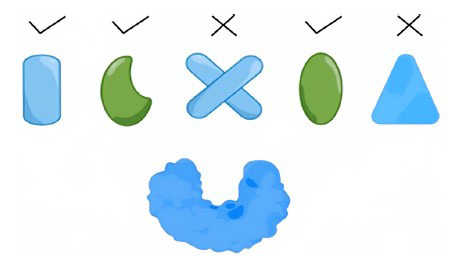
The spatial matching between the compound and the receptor protein was demonstrated by the lock-key model. This Figure is drawn by the FigDraw platform.

Based on the 3D structure of the receptor, molecular docking can automatically match the binding sites of small molecules in the compound database. In the docking process, the conformation and position of the receptor compound are constantly changed to optimize the dihedral angle of the rotatable bond in the molecule. The binding energy of possible binding modes is then calculated by scoring function according to the principles of energy, geometric and chemical environment complementarities, and finally, the energy ranking of compounds is obtained [32]. Thus, the modes with the lowest energy score can be considered the most stable ones closest to the natural combination mode [33]. The initial idea of molecular docking can be traced back to the “lock and key principle” proposed by Fisher in the 19th century, which mainly emphasizes spatial matching [34] (Fig. **5**). With the development of receptor theory, people have a deeper understanding of the interaction between physiologically active molecules and biomolecules. The conventional rigid model based on spatial matching in molecular docking has been replaced by the flexible model based on both spatial matching and energy matching. The optimization of the model greatly improves the prediction ability of molecular docking.

Molecular docking methods are mainly divided into three categories [35]: 1) rigid docking, 2) semi-flexible docking and 3) flexible docking. Rigid docking excludes changes in the receptor and ligand conformation during the docking process [36]. Rigid docking can be used to study large systems, such as protein-protein and protein-nucleic acid interactions. The calculation is simple, and the degree of fit between objects is mainly considered. Representative docking software includes ClusPro, Hex, HDOCK and ZDOCK. Semi-flexible docking is often used for the docking of small molecules and macromolecules [37]. In the docking process, the conformation of small molecules can be changed within a certain range, whereas those of macromolecules are rigid. In this way, we can not only investigate the influence of flexibility to a certain extent, but also maintain high computational efficiency. Semi-flexible molecular docking method is generally used in drug design and VS. Representative software for semi-flexible docking includes AutoDock Vina, USCF DOCK, Glide and GOLD. Fig. (**6**) shows the 3D docking results of polypyrimidine tract binding protein RRM2 and compound LDN193189. Flexible docking method is generally used to accurately study the recognition between molecules [38]. Although flexible docking method can improve the docking accuracy due to the allowance of the conformational change of the docking system, it takes a long time. Representative software for flexible docking includes Fiber Dock, Fleksy and Schrödinger suites (Table **2**).

The progress of molecular and structural biology has provided more and more important structural information of drug targets [39], making molecular docking a technical platform for efficient and rapid acquisition of compound target interaction information for innovative drug development. It is generally believed that the compound conformation with the lowest free energy is the best binding one. The common methods to search for the best conformation are the systematic search method and the non-systematic search method. The systematic search method evaluates all possible binding conformations by changing each torsion angle, and then selects the one with the lowest energy. This method requires a lot of calculation. Therefore, the non-systematic search method is usually used to find lower energy conformations. The common methods include molecular dynamics method, random search, genetic algorithm, distance geometry algorithm and so on [40]. The scoring function aims to effectively estimate the interaction energy between protein and conformation of all compounds. However, the accuracy usually has to be weakened to obtain efficiency [41]. There are three types of scoring functions: 1) force field function, 2) empirical function and 3) knowledge-based function [42]. A targeted scoring function can be designed to improve the evaluation performance of molecular docking [43].

### 2.4 Combination Method of Virtual Screening

The combination of LBVS and SBVS is a good choice when the structure of ligand-protein complex and a similar relationship with active compounds are available. It not only strengthens the complementarity of the two methods but also balances their limitations, so as to enhance the reliability of VS results. In most cases, LBVS and SBVS technologies are combined in a sequential or parallel manner [44]. The serial combination is designed as a hierarchical process through sequential filters to largely reduce the number of compound source databases. Generally, the method with convenient and fast calculation is used as the first filter, and the method with higher calculation cost and accuracy is used as the second filter to construct the search calculation to meet higher calculation requirements. Another sequence of VS based on the combination method of LBVS and SBVS is called the second phase similarity search [45], in which the compounds screened by molecular docking based on the same target are used as reference molecules. These new reference molecules and other molecules with similar structures are used as a compound library for LBVS, so as to explore the chemical field of these new hit molecules. The parallel application of LBVS and SBVS is usually used to compare the accuracy of the two methods and improve the complementarity with each other [46]. Combined applications show better advantages than a single use of SBVS or LBVS. However, reports on the combined application of SBVS and LBVS show mixed results [47]. The design and application of a more perfect combination needs further research.

### 2.5 Pharmacokinetic Properties

Although a compound can achieve good affinity with its target *in vitro*, it does not mean that desired effect *in vivo* can be achieved. Poor pharmacokinetic properties and unacceptable toxicity are the main reasons contributing to an unsuccessful clinical trial of candidate drugs [48]. Therefore, choosing appropriate candidate drugs with a good balance in absorption, distribution, metabolism, excretion and toxicity (ADMET) is a necessary prerequisite for a successful VS. ADMET is a comprehensive study on the pharmacokinetic properties and toxicity of drugs. The most common assessments involve physicochemical properties, including solubility, lipophilicity, permeability, affinity, and metabolic stability [49]. Other ADMET characteristics [50], such as human intestinal absorption (HIA), blood-brain barrier (BBB) permeability, cytochrome P450 enzyme (C) inhibition, drug-induced liver injury (DILI), cardiotoxicity and cytotoxicity. They can also be used as good indexes to evaluate candidate drugs. The most famous theory used to evaluate the bioavailability of oral drugs in history is called the “Lipinski's five rules” [51], which includes: 1) the molecular weight is less than 500, 2) the number of hydrogen bond donors is less than 5, 3) the number of hydrogen bond receptors is less than 10, 4) the lipid water partition coefficient is less than 5, and 5) the number of rotatable bonds is no more than 10. By excluding non-drug or unstable compounds, the discovery and development cycle of drugs can be shortened and the failure rate of VS can be largely reduced. At present, there are many tools that can be used for ADMET prediction, such as QikProp [52], DataWarrior [53], MetaTox [54], MetaSite [55] and StarDrop [56].

### 2.6 Experimental Validation

At present, the estimation of absolute energy of intermolecular interaction by VS algorithm does not reach satisfactory accuracy. The real interaction between small molecular compounds and targets still needs to be verified by biological experiments. However, the results of a single experiment cannot guarantee the absolute drug activity of a selected compound. Although some false-positive compounds do not specifically bind to a target protein, they may show similar drug activity due to the interference of some experimental factors [57], such as fluorescent or highly colored compounds, active metal compounds, toxic compounds and compounds coating the protein. We can use *in-silico* tools that implement chemical similarity and substructure searches to identify these false-positive compounds with specific structures, and verify the results many times by using different experimental methods [58]. Although the results of false-positive compounds are not expected, we can use false-positive compounds as a reference for similarity search in VS, so as to reduce the error of the experimental results.

## 3 NERVOUS SYSTEM DISEASES

### 3.1 Overview and Clinical Pattern of Nervous System Diseases

Neurological diseases, according to the World Health Organization, are one of the most serious dangers to public health. They can be divided into central nervous system diseases and peripheral nervous system diseases [59], such as Alzheimer's disease, Parkinson's disease, depression, epilepsy, insomnia, cerebral ischemia, brain injury, multiple sclerosis, peripheral neuropathy, multiple brain nerve injury and spinal nerve diseases. Nerve injury or degenerative diseases usually cause neuronal loss and nerve circuit injury, resulting in a series of clinical manifestations, including motor, language, social interaction, sensory and perceptual disorders, which seriously affect the health and quality of life of patients [60]. Due to the complexity of the nervous system, the methods used to treat nervous system diseases are limited. At present, the clinical treatment of nervous system diseases, such as drug therapy, gene therapy and rehabilitation training, can only improve the symptoms without fully stopping the progression of the disease [61].

In recent years, stem cell transplantation has opened up new possibilities for the treatment of nervous system diseases. Stem cell transplantation is to transplant healthy stem cells, such as mesenchymal stem cells, neural stem cells and adipose stem cells, into the human body, or locally inject them into brain lesions, where the stem cells may function in repairing central nervous system damage [62]. Although “alternative therapy” using exogenous stem cells brings hope, there are still many challenges to be solved, including cell resources, delivery strategies, cell maturation and cell integration [63].

It has been believed for many years that neurons have little regenerative ability [64]. With the in-depth research on various technologies to enhance brain neurogenesis, it has become a reality to promote the generation of new neurons in the brain. This “regenerative therapy” is an important milestone in the treatment of nervous system diseases. There are two main strategies for generating new neurons in the adult brain: 1) enhancing adult neurogenesis from neural progenitor cells [65] and 2) reprogramming non neuronal cells into neurons [66]. Neural progenitor cells in most regions of the mature central nervous system may have potential neurogenic programs, which can supplement the loss of neurons by activating their self-repair *in vivo*. Reprogramming of non-neuronal cells is also known as transdifferentiation, which has shown a good application prospect in the field of neural regeneration [67].

### 3.2 Potential Application of Virtual Screening in Neuronal Reprogramming

Transdifferentiation is an extremely complex cell reprogramming process. Cells with the transdifferential potential can change from one differentiation state to another, or differentiated cells can return to the pluripotent or totipotent state, under which their transcriptional expression profile, epigenetic modification and chromatin structure are changed [68]. Because astrocytes are close to neurons in stem cell origin and they can proliferate after brain injury, they are considered to be an ideal cell reservoir for neuronal regeneration [69]. At present, there are two main methods to induce the transdifferentiation of non-neuronal cells into functional neurons: reprogramming based on induction of transcription factors and direct reprogramming based on small molecular compounds treatment.

Transcription factor-based reprogramming is to transfer the viral vector carrying transcription factors into cells to induce transdifferentiation, which is the most commonly used transdifferentiation induction method. Wu *et al.* designed an *in vivo* cell transformation technique to reprogram striatal astrocytes into GABAergic neurons in R6/2 and yac128 HD mouse models through AAV-mediated ectopic expression of NeuroD1 and Dlx2 transcription factors [70]. Pang's team observed in previous studies that the forced expression of three transcription factors, Brn2, ascl1 and myt1l, can successfully transform fibroblasts from mice into functional neuronal cells [71]. Subsequent studies have proved that the same three transcription factors can induce human pluripotent stem cells to differentiate into functional neurons 6 days after transgene activation [72]. Guo *et al.* directly reprogrammed the reactive glial cells in the cortex of Alzheimer's disease (AD) model mice into glutamatergic neurons *in vivo* by expressing a single neural transcription factor NeuroD1 through retrovirus [73]. Qian *et al.* reported a method of transforming mouse and human astrocytes into functional neurons by temporarily inhibiting the expression level of ptbp1 using antisense oligonucleotides, and cell reprogramming and activation of endogenous neural circuits were achieved in mice by the same method [74].

Compared with reprogramming based on transcription factors, reprogramming technology based on small molecular compounds has a wider range of applications. Small molecular compounds are not only easy to synthesize, but also have good cell permeability and plasticity in biological effects. Cell reprogramming induced by small molecular compounds has the advantages of simple operation, low cost and good medicinal property [75]. It avoids the problem of host gene mutation caused by the introduction of transcription factors. Currently, most scientific researchers use a mixture of small molecular compounds to induce cell transdifferentiation. Cheng *et al.* transformed astrocytes into neuronal cell morphology in the form of the mixture using small molecular compounds VPA, CHIR99021 and Repsox or Tranilast, and these transformed neuronal cells spontaneously showed postsynaptic current [76]. Gao *et al.* Transformed astrocytes into neurons using a combination of small molecular compounds (VAP, chir99021, repsox, forskolin, i-bet151 and isx-9), which can survive and mature electrophysiologically when transplanted into the brain of postnatal mice [77]. Hu *et al.* screened small molecular compounds (VPA, chir99021, repsox, forskolin, sp600125, g06983 and Y-27632) to directly induce fibroblasts from normal people and patients with AD into glutamatergic neurons. The transdifferentiation efficiency of fibroblasts from patients with AD is similar to that of normal human fibroblasts, However, when compared to human chemical-induced neuronal cells (hciNs) derived from healthy people, the extracellular Aβ42 level and the Aβ42/ Aβ40 ratio were higher in hciNs derived from fibroblasts derived from patients with familial Alzheimer's disease (FAD) carrying mutations in human amyloid precursor protein (APPV717I) [78].

Virtual screening provides a good technical platform for obtaining small molecular compounds that can induce cell reprogramming. For the application of LBVS technology, small molecular compounds can be collected through the following four strategies [79]: 1) consulting the literature and relevant databases to collect chemicals that are similar to drugs used in clinical trials for neuronal reprogramming or small molecular compounds with similar mechanisms, 2) identifying compounds that produce similar transcriptional reactions to these drugs, 3) searching for compounds that can act on proteins or transcription factors that mediate neuronal reprogramming, which may have potential properties to promote neuronal transdifferentiation, and 4) exploring the gene transcriptional changes in the process of neuron reprogramming through bioinformatics technology mining or RNA-seq, determining compounds that can trigger these transcriptional changes, and then establishing pharmacophore model or QSAR model to search for the corresponding database to obtain the best compound molecules, and optimize and transform the structure of the obtained compound molecules, and construct new compounds with similar efficacy. For the application of SBVS technology, transcription factors and proteins related to neuronal reprogramming can be collected, and small molecular compounds with potential neuronal reprogramming effects can be collected by the above methods by consulting literature, and then simulating the interaction between small molecular compounds and proteins through molecular docking. Small molecular compounds with cell transdifferentiation promotion potential by regulating related proteins or transcription factors can thus be determined.

Although cell reprogramming induced by small molecular compounds brings hope for the treatment of neurological patients, the efficiency of small molecular compounds in inducing cell transdifferentiation needs to be improved due to the low specificity, which is also the hotspot and difficulty of small molecular compounds screening [80]. In addition, the intrinsic mechanism of cell reprogramming needs to be further clarified, so as to screen small molecular compounds or other substances with more safety, reliability and higher induction efficiency to provide effective cell resources for disease treatment. At present, the research on induced cell reprogramming mainly focuses on human and mouse species, and there is less research on other large mammals such as domestic animals, pigs, cattle and sheep, which is also a field to be explored in the future [81].

## 4 RECENT ADVANCES AND SUCCESSFUL EXAMPLES OF VIRTUAL SCREENING IN NERVOUS SYSTEM DISEASES

### 4.1 Application of Virtual Screening in Alzheimer’s Disease Treatment

Alzheimer's disease is a neurodegenerative disease. Its clinical manifestations include cognitive dysfunction, abnormal mental behavior, decreased intelligence and decreased ability to live. AD is caused by extracellular β amyloid plaque deposition (Aβ), neurofibrillary tangles (NFTs) formed by hyperphosphorylated intracellular tau protein and the loss of central cholinergic neurons [82]. The causes of AD are also related to age, genetics, environment, oxidative stress, inflammation and other factors. At present, the drugs against AD include cholinesterase inhibitors, such as donepezil, rivastigmine and galantamine, which can delay the decomposition of acetylcholine within the synaptic space [83], and the NMDA receptor antagonist memantine, which can reduce the excitotoxicity and protect neuronal cells [84]. Most of these drugs function by relieving symptoms, and efficient treatments against AD is still unavailable in the present.

Cholinesterase is especially suitable for drug discovery through VS as an AD target due to its well-characterized active sites and thorough knowledge of the structure−activity relationships of existing inhibitors [85]. Bajda *et al.* aimed to screen new acetylcholinesterase inhibitors (AChEI) through SBVS in the non-imidazole histamine H3 receptor-ligand library. 26 compounds that may bind to the catalytic and peripheral sites of acetylcholinesterase and cholinesterase were screened by molecular docking. Subsequently, the inhibitory effects on acetylcholinesterase (AChE) and butyryl cholinesterase (BuChE) were evaluated in Ellman's analysis. The most effective two compounds were selected. The kinetic study showed that they were non-competitive inhibitors of AChE and BuChE. Compounds 5 (2-(4-(6-(Azepan-1-yl) hexyloxy) phenyl)-6-methyl-4H-chromen-4-one hydrogen oxalate) and 17 (6-Methyl-2-(4-(6-(piperidin-1-yl)hexyloxy)-phenyl)-4H-chromen-4-one hydrogen oxalate) can be useful as lead structures in the development of novel multi-target anti-AD disease medicines [86]. Lu *et al.* automatically generated 4910 BChE pharmacophore models through the discovery studio (DS) 3.0 software, selected the pharmacophore model with the best performance after verification by ROC curve, and conducted VS on six compound databases (chemdiv, druglikediverse, enamine, interbioscreen, PPI and specs database). Molecules that passed drug-like filters were further studied by docking and cluster. Finally, 17 potential targets were selected and their ability to inhibit BChE from equine serum (eqBChE) and AChE from electrophorus electricus (eeAChE) was tested at a concentration of 10 μM. The experimental results showed that 8 compounds exhibited BChE inhibition activity of more than 50.0% and showed selective BChE inhibition activity. These compounds undoubtedly provide a good basis for the discovery of highly selective BChE inhibitors [87].

### 4.2 Application of Virtual Screening in Parkinson’s Disease Treatment

Parkinson's disease is a central nervous system disease caused by the reduction of dopamine content in the striatum due to the deformation of dopaminergic neurons in the substantia nigra (SN) of the midbrain [88]. The clinical symptoms of PD mainly include motor symptoms such as tremor, myotonia, motor retardation and abnormal posture and gait, and non-motor symptoms such as sleep disorder, depression and anxiety. PD is the second most common neurodegenerative disease after AD [89]. The pathogenesis of PD remains unclear. It is generally believed to be related to abnormal protein aggregation, mitochondrial dysfunction, oxidative stress and so on. Clinically, the commonly used treatment methods of PD include drug treatment supported by levodopa, surgical therapy and physical therapy [90].

Levodopa is usually used to supplement the dopamine level of the central nervous system. Levodopa is converted to dopamine by dopa decarboxylase (DDC). Peripheral DDC inhibitors can inhibit the activity of peripheral DDC and make more levodopa enter the brain to play a role [91]. Based on the crystal structure of DDC carbidopa complex, Daidone *et al.* screened the compounds with the highest binding fraction in the public database through the VS method of pharmacophore model and molecular docking combination, and tested their ability to bind and inhibit the purification of recombinant human DDC. Compounds with the highest inhibitory activity were used for the second similarity-based filtering of the public zinc database to retrieve analogues, thereby expanding the new category of DDC inhibitors. *In vitro* experiments showed that the nine compounds screened from the second screening inhibited human DDC in a competitive mode, and the K(i) value was within the range of 2-15 mM. Subsequently, by using the core of the most active compound for substructure search, a molecule with a K(i) value of 500 nM became a new lead compound targeting DDC [92].

Monoamine oxidase (Maos) is an enzyme responsible for the metabolism of monoamine neurotransmitters, which can regulate the concentration of dopamine in both central and peripheral nervous system tissues [93]. Crisan *et al.* detected new natural products (NPS) of potentially selective MAO-B inhibitors and calculated the therapeutic effect of repositioned listed drugs on PD. They used 3D similarity method to conduct retrospective calibration experiments on selective MAO-B data sets to determine the most appropriate similarity coefficient and estimate its related threshold for prospective screening. The consensus scheme was further used to conduct prospective 3D similarity search on specs NP and drugbank data sets. The screening results revealed that cardamom possesses potential selectivity for MAO-B active sites. Through ADMET verification, it is found that it has good pharmacological properties. Two marketed drugs, finamisol and benzophenone, are considered promising candidates for the treatment of PD [94].

### 4.3 Application of Virtual Screening in Epilepsy Treatment

Epilepsy is a chronic brain disease resulting from a number of factors. It is characterized by sudden, repeated and transient dysfunction of the central nervous system caused by excessive discharge of brain neurons [95]. Epilepsy has complicated pathophysiology that has yet to be fully understood. The imbalance between excitation and inhibition of neuronal activities in the central nervous system is generally considered the underlying mechanism. In recent years, studies on the pathogenesis of epilepsy have shown that this imbalance between excitation and inhibition is mainly related to the changes in ion channels, synaptic transmission and connection, neurovascular units and glial cells [96].

Anticonvulsant drugs (ACD) are the main antiepileptic drugs [97]. Palestro *et al.* aimed to develop new anticonvulsant drugs for the treatment of refractory epilepsy. They assembled the human NaV1.2 three-dimensional model, and the compounds, after preliminary screening through molecular docking, can then be screened in the second stage. They constructed and established the 3D structure model of human P-gp, and screened the docking results again. The compounds with high screening fraction were tested on the NaV1.2 channel by patch-clamp technique, and the MES-test was carried out *in vivo*. Patch-clamp studies confirmed that candidate drugs such as ciprofloxacin, losartan, valsartan and synthetic compound N.N´-diphenethylsulfamide inhibited the activity of NaV1.2 channel. The MES-test confirmed that valsartan, ciprofloxacin and N,N´-diphenethylsulfamide can exert antiepileptic effects [98].

Important genetic evidence suggests that a series of idiopathic generalized epilepsy syndromes are associated with γ-Aminobutyric acid - A (GABAA) receptor mutation [99], which is a promising target for the development of new potential antiepileptic drugs. Mehta *et al.* used the crystal structure of a subtype-selective β3 homopentameric ligand-gated ion channel of GABAA receptor for the first time, screening for GABAA agonists as potential antiepileptic drugs in the Asinex library. The top-ranked compounds were further screened by binding energy estimation, ADMET analysis and ligand efficiency matrix calculation. Most of the screened compounds have a three-carbon gap between the amino group and morpholine ring, which is similar to the reported structure of GABAA orthosteric agonists. According to the predicted structure-activity relationship (SAR), it can be proposed that a series of 4-(3(ethylammonio)propyl)morpholin-4-ium scaffold molecules with specific structural characteristics have potential antiepileptic activity by targeting GABAA [100].

### 4.4 Application of Virtual Screening in Depression Treatment

Depression is a common mental disorder characterized by significant and lasting depression. Despite the fact that neuroscience research has progressed significantly over the last few decades, the pathophysiology of depression has not been fully clarified. Studies have revealed several mechanisms, including changes in serotonergic, noradrenergic, dopaminergic and glutamatergic systems, increased inflammation, vascular changes, structural and functional brain changes, neuroplasticity and neurogenesis [101]. At present, the monoamine neurotransmitter hypothesis has been widely accepted. Depression is believed to be caused by changes in the levels of one or more monoamines, including serotonin, norepinephrine and dopamine. Most of the common antidepressants in the clinic are based on the above hypothesis, including tricyclic antidepressants, monoamine oxidase inhibitors and selective serotonin reuptake inhibitors [102].

The decrease of monoamines, such as that of serotonin, in the brain, is considered to be the main cause of depression [103]. The plant extract of the mesodermal alkaloid family (Aizoaceae) has the characteristics of serotonin reuptake inhibitor in pharmacology because it contains mesodermal alkaloids. Said *et al.* aim to evaluate the antidepressant activity of the alkaloidal fraction of the roots of Mesembryanthemum cordifolium. The roots of mesembryanthemum cordifolium were studied by silicon-based and structure-based screening, and the structural similarity between candidate compounds and FDA approved antidepressants was analyzed by “Swiss Similarity” online software. Then, the antidepressant activity of compounds with high scores was detected by forced mouse swimming test. The results showed that the antidepressant activity of the alkaloid component was better than that of standard antidepressant imipramine hydrochloride. This may be partly due to the fact that it contains a considerable number of membrane alkaloids, which can effectively inhibit the activity of serotonin [104].

Increasing the concentration of 5-hydroxytryptamine (5-HT) in the synaptic space by inhibiting serotonin transporters (SERT) has been reported to improve psychological and neurological disorders [105]. Based on the recently discovered crystal structure of SERT, a combination of high-throughput virtual screening and docking was used to screen new candidate SERT inhibitors at the central and allosteric binding sites of SERT from the OTAVA chemical database. Four potent compounds with high scores (160234, otava ID: 7118020138; 159166, otava ID: 7117171303; 69419, otava ID: 118671819) contain the central binding site (S1), and one compound (93507, otava ID: 6248262) contains the allosteric binding site (S2). Molecular dynamics (MD) simulation is then used to clarify their structure and dynamic distribution in SERT binding cavity. These four compounds were compared with the central and allosteric binding site inhibitors paroxetine (8pr) and escitalopram (68p), and the binding free energy calculation (mm / GBSA) also confirmed that the identified compounds have high binding power to SERT [106].

### 4.5 Application of Virtual Screening in Spinal Cord Injury Treatment

Spinal cord injury (SCI) refers to the direct injury of the spinal cord caused by direct or indirect external factors, which results in varied degrees of loss of motor and sensory abilities. Violence and accidents are the main causes of SCI [107]. Spinal cord injury mainly includes primary mechanical injury and subsequent secondary injury caused by microcirculation disorder and excessive inflammatory reaction [108]. Methylprednisolone is the only drug widely used in the treatment of primary SCI, although its curative effect is limited and has obvious side effects [109]. At present, the research focus is to prevent secondary SCI by changing neuroinflammation [110], reducing free radical injury [111], relieving excitotoxic injury to neurons [112], improving blood flow [113] and counteracting ion exchange effect at the primary injury site [114].

Mature oligodendrocytes undergo extensive cell death in the injured spinal cord, thus destroying white matter repairs and axonal integrity [115]. Previous studies have shown that inhibition of protein tyrosine phosphatase sigma (PTPσ) and leukocyte common antigen-related phosphatase (LAR) can promote the preservation and renewal of oligodendrocytes. Zhou *et al.* used VS, molecular docking and molecular dynamics simulation to explore potential dual-target drugs for the treatment of SCI. The results showed that zinc72417086 showed a higher docking score and met Lipinski's five laws. The post-analysis demonstrated that when ZINC72417086 is bound to PTPσ and LAR, it can stabilize the protein conformation and destroy the residue interaction between P-loop and other loop regions in an active pocket. In the meantime, residues ARG1595 and ARG1528 within ZINC72417086 can play a crucial role in the inhibition of PTPσ and LAR [116].

Recent studies have shown that the upregulation of the Ras/Raf/extracellular signal-regulated kinase 1/2 (ERK1/2) signaling pathway is related to the occurrence of SCI [117]. Yan *et al.* selected about 5000 compounds in the Interbioscreen natural compound database for VS to identify new Ras small molecule inhibitors. The compounds with higher docking scores were further screened according to Lipinsky's five rules, and a total of five compounds were calculated. These compounds can dock with the GTP binding site of Ras, and the inhibition of this site is considered to be an important means to down-regulate Ras/Raf/ERK1/2 signaling pathway. The identified lead compounds were then simulated by molecular dynamics. The results showed that lead 3 ((13S)-13-methyl-6,7,8,9,11,12,13,14,15,16- decahydrospiro[cyclopenta[a]phenanthrene-17,2'-[1,3]dioxolan]-3-ol) had the best blood-brain barrier (BBB) permeability and plasma protein binding (PPB), as well as the lowest ΔG value. The stability and binding mode of the binding site between lead3 and GTP binding site of Ras were further explored by 100 nsec MDS test. The results showed that GLY60 in the GTP binding site of Ras was the best binding site during 100 nsec operation [118].

### 4.6 Application of Virtual Screening in Amyotrophic Lateral Sclerosis (ALS) Treatment

Amyotrophic lateral sclerosis (ALS) is a devastating neuromuscular degenerative disease with an incidence rate of 2/100000. According to information about the involvement of family history, ALS is divided into familial amyotrophic lateral sclerosis (fALS) and sporadic amyotrophic lateral sclerosis (sALS) [119]. The typical clinical manifestations are progressive spasticity, hyperreflexia, myasthenia and muscular atrophy. ALS patients gradually grow feeble due to the selective loss of upper and lower motor neurons, which eventually leads to death [120]. The pathogenesis of ALS is very complex, and the mechanisms affect each other. The main pathogenesis theories include gene mutation, neuroexcitotoxicity, mitochondrial abnormalities, oxidative stress, immune-inflammatory response and so on. At present, there is no effective treatment for ALS. The internationally recognized therapeutic drug for ALS is riluzole, a benzothiazole glutamate antagonist. However, its effect is limited and it cannot reverse the pathological changes of damaged motor neurons [121].

One of the four primary mutation clusters in the development of ALS is mutations in the gene that encodes copper/ zinc superoxide dismutase (SOD1) [122]. Therefore, the development of a proton pump inhibitor (PPI) of SOD1 can be used as a new therapy for ALS. Previous studies have shown that tubulin can interact with N-terminal residues 1-23 of mutant SOD1. Hirayama *et al.* used the established mutant SOD1 tubulin interaction inhibition analysis system as the internal model system to screen 32791 compounds for mutant SOD1 PPI inhibitors through molecular docking. Five of the compounds with higher docking fractions inhibited the mutant SOD1-tubulin interaction *in vitro*. Binding pattern analysis predicted that some inhibitors may bind to the tubulin binding site of g85r SOD1 through π electron interaction with the aromatic ring of trp32 residue of g85r SOD1 [123].

In order to further explore the effect of SOD1 toxicity on ALS, DuVal *et al.* verified that the tryptophan residue at position 32 (W32) is involved in SOD1 misfolding in the morphological and functional model of zebrafish motoneuron axon. Therefore, drugs can be designed to target this residue to improve the symptoms of ALS. Similar compounds were screened using pharmacophore models based on the molecular characteristics of the uracil moiety of small molecules previously predicted to interact with W32. Finally, telbivudine was selected for biological verification after ADMET analysis. It was found that nucleoside telbivudine significantly saved SOD1 toxicity in a dose-dependent manner in the neuromuscular damage model. Therefore, W32 residue has an impact on SOD1 toxicity *in vivo*, making it a promising target for future therapeutic research [124].

### 4.7 Application of Virtual Screening in Multiple Sclerosis (MS) Treatment

Multiple sclerosis (MS) is a chronic inflammatory and demyelinating disease of the central nervous system (CNS). Typical syndromes include monocular vision loss caused by optic neuritis, limb weakness or sensory loss caused by transverse myositis, diplopia caused by brainstem dysfunction or ataxia caused by cerebellar lesions [125]. Genetic infection, dietary habits, vitamin deficiency, smoking and intestinal microbiota imbalance are considered to be involved in the pathogenesis of MS. Immunological pathogenesis is one of the widely recognized main pathogenesis of MS, which is mainly the irreversible axonal injury caused by the entry of activated autoreactive T lymphocytes in peripheral blood into the central nervous system (CNS) [126]. At present, more than ten kinds of drugs can be used to reduce the frequency of transient neurological dysfunctions caused by MS. Among the disease-modifying agents (DMAS), interferon (IFN) β and Glatiramer acetate are the first-line drugs for the treatment of recurrent MS. However, no drug can completely prevent or reverse the deterioration of progressive neurological functions caused by MS [127].

Recent studies have shown that inhibition of Rho kinase-2 plays an important role in the treatment of MS [128]. Shah *et al.* used the GALAHAD module of Sybyl to generate the pharmacophore model of Rho kinase-2 inhibitors with 8 different structures. The best model contains 2 hydrophobic atoms, 1 donor atom and 2 receptor sites. The model is verified by GH and ROC methods. Forty compounds were screened by molecular docking in the compound library, and the properties of ADMET were analyzed, suggesting RIK 16 (N-(benzo[d]thiazol-2-ylmethyl)-4chloropyridin-2-amine) to be a promising molecule as Rho kinase-2 inhibitor [129].

Studies have shown that the activation of SIP1 receptor can lead to receptor internalization and degradation, so as to prevent the auto reactivity of immune cells in the target tissues [130]. Therefore, SIP1 receptor agonists can be a suitable candidate for the treatment of autoimmune diseases such as MS. Based on the structure of the SIP1 receptor, Alizadeh *et al.* established 3D-QSAR predictive models for 62 compounds containing 2-imino-thiazolidine-4-1 derivatives. Using the generated model, the selected compounds were virtually screened for SIP1 receptor agonists in the PubChem chemicals database. The results showed that four potential compounds with high potency and selectivity for the S1P1 receptor were obtained. The affinity between the selected compounds and S1P1 receptor was evaluated by MD simulation method. The results indicated that the binding energies of the compounds were within the range of -39.31 to -46.18 and -3.20 to -9.75 kcal/mol, as calculated by MM-GBSA and MM-PBSA algorithms, respectively [131].

## 5 LIMITATIONS OF VIRTUAL SCREENING

Virtual screening is mainly divided into SBVS and LBVS. Compared with LBVS, SBVS does not rely on the information of known ligand molecules. It can screen novel drugs with unique action mechanisms completely based on the characteristics of the receptor, but this strategy depends on the 3D structure of the receptor [132]. When the target to be studied lacks the crystal structure determined by nuclear magnetic resonance (NMR) examination, its application is limited. Due to the advance of homology modeling technology in recent years, researchers can find proteins that are homologous with the target sequence and have experimentally determined structures as templates to construct the structural model of the target sequence on the basis of sequence similarity analysis. However, its accuracy depends on the quality of the template and the accuracy of key steps [133]. In addition, the number of protein structures that may be utilized as templates is limited, which also limits the application of homology modeling. There are numerous elements that play a part in the drug binding process, which has not been fully considered in the current calculation theory and calculation model. Thus, the current computer VS method cannot achieve completely ideal screening results. However, if more complex and vital energy factors are considered in the early stage of VS, the computational cost will increase greatly, thus reducing the efficiency and limiting the application of VS technology.

To ensure efficiency while comprehensively considering other higher-level energy factors to improve the prediction ability of VS remains the main challenge in its current application. The commonly used scoring function has low accuracy on the basis of wide applicability, and cannot reliably predict the binding strength of ligands in all protein systems [134]. The best scoring function is usually a targeted scoring function, which can meet the needs of interested goals. Another way to improve the accuracy of the scoring function is consensus scoring. These schemes combine different scoring functions, which are meant to counteract the inherent errors of various scoring indicators [135].

Reducing false positivity is still the key problem in the practical application of VS technology in drug discovery. The compound was screened by the false-positive compound exclusion method. Generally, compounds with false-positive characteristics are easy to decompose under hydrolysis conditions and can react chemically with non-targeted proteins and biological nucleophiles. However, in special cases, this exclusion method is not applicable if the compound showing a certain chemical reaction with biological macromolecules is the target of drug screening and discovery. Compared with SBVS, LBVS technology is limited by existing active molecules, and it lacks structural novelty and uniqueness of action mode. More importantly, it is difficult to detect a new drug target using this method if there are no known ligand molecules to obtain for model construction. In this case, researchers usually need to resort to receptor-based methods to find innovative inhibitor molecules [136]. However, the LBVS method does not rely on the energy calculated by the scoring function to evaluate the quality of compound molecules, so as to avoid interference with the screening results due to the defect of accurate calculation of free energy. Thus, the combination of LBVS and SBVS may achieve complementary advantages.

## CONCLUSION AND FUTURE PERSPECTIVES

VS reduces the number of target data sets by applying different methods and step-by-step filters, so as to screen compounds with potential medicinal value. The active sites of specific target molecules are used to construct a new chemical skeleton to enrich the target data set. In this review, we introduce the main methods of VS and their application in the prevention and treatment of nervous system diseases. Due to the complexity of nervous system diseases, the limited financial resources and long research period in the development of entity drugs undoubtedly bring many difficulties to researchers. It is thus hard to determine a definite target for VS. However, we can re-evaluate the potential pathogenesis of nervous system diseases on the basis of consulting previous studies. In addition, we can take the relevant targets of the neural network as the reference mold, create efficient matching filters, and then use VS to screen compounds that may be related to the disease phenotype and bind to multiple targets. Organically combining the establishment of a drug entity database with VS platform can effectively expand the chemical space of drugs for the treatment of nervous system diseases and maximize the efficiency of VS-based compound screening research.

Although VS plays a key role in drug discovery, much work is needed to improve the training method and thus enhance its application in screening drugs against central nervous system diseases. Both receptor-based strategies and ligand-based methods have some obvious shortcomings. VS only simulates the process of high-throughput screening on the computer, so its performance does not depend on the computer method, but mainly depends on the identification of drug action and the theoretical guidance developed from it. At present, due to the deficiency in understanding and clarification of the mode of drug actions, the existing calculation technology and simulation means cannot ensure the absolute correctness and reliability of the calculation results. Virtual screening technology is now in a mature stage of development, but it is far from perfect. With the continuous exploration of computer-aided drug theory and the emergence of various emerging technologies, such as new prediction algorithms based on machine and deep learning, their combined application with VS will greatly increase the success rate of VS and improve the quality of screening results. We have good reason to believe that VS will facilitate us to gain more efficient drugs in the treatment of nervous system diseases.

## Figures and Tables

**Fig. (2):**
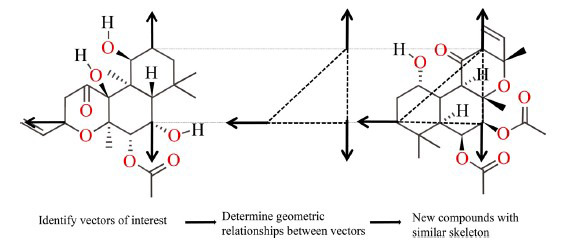
A method for identifying similar 2D skeleton. Take the structure of Forskolin and Forskolin G as an example.

**Fig. (3):**
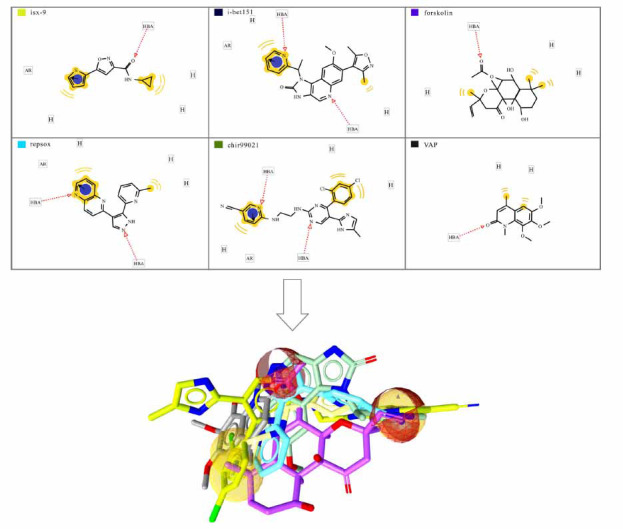
Construct a ligand-based pharmacophore model that may promote the transdifferentiation of astrocytes. The ligands-based pharmacophore model was constructed by using the ligscout module of discovery Studio software. VAP was the test set and the other five compounds were the training set. It has been reported that the mixture of these six compounds can induce the transformation of astrocytes into neurons. This pharmacophore characteristics include hydrogen bonds (yellow) and π-π conjugation (red).

**Fig. (4):**
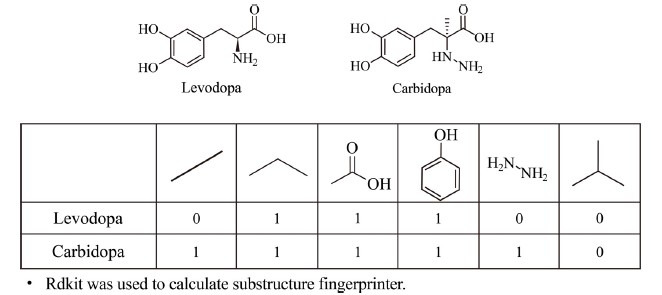
2D-substructure fingerprints of levodopa and carbidopa. In binary representation, the number 1 indicates the presence of a feature and the number 0 indicates the absence of a feature.

**Fig. (6):**
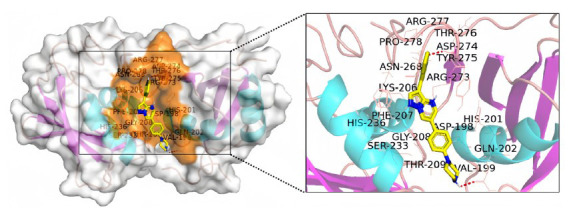
3D docking results of polypyrimidine tract binding protein RRM2 and compound LDN193189. AutoDock software was used to perform this molecular docking. It shows that there are two polar contacts (red) between polypyrimidine tract binding protein RRM2 and compound LDN193189.

**Table 1:** Common databases for virtual screening.

**Library**	**Description**	**No. of Compounds ** **and/or Targets**	**Availability**	**Website**
Pubchem	A public database of biological activity data resources of small organic molecules.	111566735 compounds and 185291 targets	Public	https://pubchem.ncbi.nlm.nih.gov/
ChemBridge	The database provides target-focused compounds, synthetic macrocycles and chemical building blocks.	1.3 million compounds	Commercial	https://www.chembridge.com
DrugBank	A database of drugs and drug targets based on the combination of bioinformatics and chemical information resources.	500000 drugs and 29131 targets	Public	https://go.Drugbank.com/
ZINC15	The database is a public access database that combines biology and chemical informatics.	750 million compounds	Public	https://zinc.docking.org/
ChemDiv	The database provides services such as the establishment, storage and management of drug screening database for multiple screening plans.	1.6 million compounds	Commercial	https://www.chemdiv.com/products/screening-libraries/
PDB	The database provides 3D shapes of proteins, nucleic acids, and complex assemblies.	193760 targets	Public	https://www.rcsb.org/
LigandBox	The database is a small molecule compound library for virtual docking.	4 million compounds	Public	http://ligandbox.protein.osaka-u.ac.jp/ligandbox/
SuperTarget	The database also integrates information related to drug pointers, adverse reactions, pathways and GO terms.	195770 compounds and 6219 targets	Public	http://insilico.charite.de/supertarget/index.php
Life Chemicals	The molecular weight of the database is good and can only be downloaded after registration.	2.5 million compounds	Commercial	http://www.lifechemicals.com/
Specs	The database is cheap and is the first database to enter the Chinese market.	350000 compound library	Commercial	http://www.specs.net/
Enamine	The database includes target database, fragment database and diversity database.	22.7 billion compounds	Commercial	http://www.enamine.net/
Maybrige	The database provides downloads of ion channels, kinases, fragments and PPI libraries.	54836 compounds	Commercial	http://www.maybridge.com
BindingDB	Structure search over 24000 Ki and IC_50_ measurements from over 10000 molecules.	1093579 compounds and 8821 targets	Public	https://www.bindingdb.org/bind/index.jsp
KEGG DRUG	The database provides chemical structure, therapeutic target, metabolizing enzyme and molecular interaction network information.	11938 targets	Public	https://www.genome.jp/kegg/drug/
ChemSpider	The database has the richest single chemical information online resources.	114 million compounds	Public	http://www.chemspider.com/
HIT	A unique database of herbal compounds and targets.	1237 herbal compounds and 2208 biological targets	Public	http://www.badd-cao.net:2345/

**Table 2:** Docking software.

**Software**	**Docking Type**	**Free of ** **Academics**	**Source**
ClusPro	Rigid docking	Yes	https://cluspro.bu.edu/login.php
Hex		Yes	http://hex.loria.fr/
HDOCK		Yes	http://hdock.phys.hust.edu.cn/
ZDOCK		Yes	https://zdock.umassmed.edu/
AutoDock	Semi-flexible docking	Yes	http://autodock.scripps.edu/
AutoDock Vina		Yes	http://vina.scripps.edu/
Glide		No	https://www.schrodinger.com/glide
GOLD		No	https://www.ccde.cam.ac.uk/solutions/csd-discovery/components/gold/
DOCK		Yes	http://dock.compbio.ucsf.edu/
Fiber Dock	Flexible docking	Yes	https://www.toptica.com/products/optical-isolators-photonicals/photonicals/fiberdock/
Flexdock		Yes	http://forge.scilab.org/index.php/p/flexdock/
Schrödinger suites		No	https://www.schrodinger.com/

## LIST OF ABBREVIATIONS

ACD: Anticonvulsant Drugs
ACFs: Atom Center Fragments
AChE: Acetylcholinesterase
AD: Alzheimer's Disease
BBB: Blood-brain Barrier
BuChE: Butyryl Cholinesterase
CNS: Central Nervous System
DILI: Drug-induced Liver Injury
DS: Discovery Studio
HIA: Human Intestinal Absorption
HTS: High-throughput Screening
LBVS: Ligand-based Virtual Screening
MS: Multiple Sclerosis
NFTs: Neurofibrillary Tangles
NMR: Nuclear Magnetic Resonance
QSAR: Quantitative Structure-activity Relationship
QSPR: Quantitative Structure-property Relationship
SBVS: Structure-based Virtual Screening
SN: Substantia Nigra
VL: Virtual Library
VS: Virtual Screening
